# Population‐specific effect of *Wolbachia* on the cost of fungal infection in spider mites

**DOI:** 10.1002/ece3.6015

**Published:** 2020-03-28

**Authors:** Flore Zélé, Mustafa Altıntaş, Inês Santos, Ibrahim Cakmak, Sara Magalhães

**Affiliations:** ^1^ Centre for Ecology, Evolution and Environmental Changes (cE3c) Faculdade de Ciências da Universidade de Lisboa Lisboa Portugal; ^2^ Department of Plant Protection Faculty of Agriculture Adnan Menderes University Aydin Turkey

**Keywords:** antibiotic treatment, bacterial community, facilitation, fungi‐induced mortality, symbiont‐mediated protection, *Tetranychus urticae*

## Abstract

Many studies have revealed the ability of the endosymbiotic bacterium *Wolbachia* to protect its arthropod hosts against diverse pathogens. However, as *Wolbachia* may also increase the susceptibility of its host to infection, predicting the outcome of a particular *Wolbachia*‐host–pathogen interaction remains elusive. Yet, understanding such interactions and their eco‐evolutionary consequences is crucial for disease and pest control strategies. Moreover, how natural *Wolbachia* infections affect artificially introduced pathogens for biocontrol has never been studied. *Tetranychus urticae* spider mites are herbivorous crop pests, causing severe damage on numerous economically important crops. Due to the rapid evolution of pesticide resistance, biological control strategies using entomopathogenic fungi are being developed. However, although spider mites are infected with various *Wolbachia* strains worldwide, whether this endosymbiont protects them from fungi is as yet unknown. Here, we compared the survival of two populations, treated with antibiotics or naturally harboring different *Wolbachia* strains, after exposure to the fungal biocontrol agents *Metarhizium brunneum* and *Beauveria bassiana*. To control for potential effects of the bacterial community of spider mites, we also compared the susceptibility of two populations naturally uninfected by *Wolbachia*, treated with antibiotics or not. In one population, *Wolbachia*‐infected mites had a better survival than uninfected ones in absence of fungi but not in their presence, whereas in the other population *Wolbachia* increased the mortality induced by *B. bassiana*. In one naturally *Wolbachia*‐uninfected population, the antibiotic treatment increased the susceptibility of spider mites to *M. brunneum*, but it had no effect in the other treatments. These results suggest that natural *Wolbachia* infections may not hamper and may even improve the success of biological control using entomopathogenic fungi. However, they also draw caution on the generalization of such effects, given the complexity of within‐host–pathogens interaction and the potential eco‐evolutionary consequences of the use of biocontrol agents for *Wolbachia*‐host associations.

## Graphical abstract

This study shows that *Wolbachia* can increase spider‐mite susceptibility to pathogenic fungi, suggesting that this endosymbiont may not hamper, but instead improve the success of fungal‐based pest control. However, this study also highlights the complexity of such interactions and draws caution on the generalization of these findings.

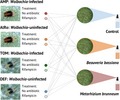

## 1 INTRODUCTION

The maternally inherited bacterium *Wolbachia* is to date the best studied and probably the most common endosymbiont of arthropods. It is estimated to infect up to 52% of arthropod species (Weinert, Araujo‐Jnr, Ahmed, & Welch, 2015), a success mainly attributed to its ability to induce various types of reproductive manipulation in hosts to increase the reproductive success of infected females, thereby increasing its own transmission (Werren, Baldo, & Clark, 2008). In particular, the ability of *Wolbachia* to spread rapidly within and among host populations (Engelstadter & Hurst, 2009) has raised growing interests in using it in biocontrol programs (Bourtzis et al., 2014).

Possible *Wolbachia*‐based biocontrol strategies include the use of *Wolbachia* as a microbial biocontrol agent, for instance to enhance productivity of natural predators and parasites such as parasitoids (e.g., Grenier et al., 1998; Stouthamer, 1993); as a potential gene‐drive vehicle for population replacement strategies through cytoplasmic drive (which provides a mechanism for the autonomous spread of desired genes into targeted populations; e.g., Dobson, 2003; Sinkins & Godfray, 2004; Turelli & Hoffmann, 1999); or for sterile insect techniques (SIT) to suppress target pest populations by repeated sweeps with infected individuals (Calvitti, Marini, Desiderio, Puggioli, & Moretti, 2015; Zhang, Lees, Xi, Gilles, & Bourtzis, 2015; Zhong & Li, 2014). Subsequently, the discovery of the ability of *Wolbachia* to protect its hosts against a wide array of pathogens, including viruses, protozoan parasites, fungi, or pathogenic bacteria (reviewed by Cook & McGraw, 2010) has provided new avenues for the control of vector‐borne diseases (reviewed by Iturbe‐Ormaetxe, Walker, & Neill, 2011). For instance, deliberate introductions of *Wolbachia* into *Aedes aegypti* mosquito populations are currently being undertaken successfully in several regions worldwide to control dengue virus (e.g. Hoffmann et al., 2014; Nguyen et al., 2015). However, such ability of *Wolbachia* to interfere with diverse host pathogens may have undesirable effects on biocontrol strategies if, for instance, natural *Wolbachia* infection interferes with parasitic biocontrol agents, a possibility that has never been addressed. Alternatively, natural *Wolbachia* infections in several host species may also increase host susceptibility to parasite infection (e.g., Graham, Grzywacz, Mushobozi, & Wilson, 2012; Hughes, Rivero, & Rasgon, 2014), raising the possibility that *Wolbachia* could also facilitate the action of biocontrol agents. Moreover, potentially variable effects of *Wolbachia* on host susceptibility to biocontrol agents may have ecological and epidemiological consequences. For instance, artificially introduced pathogens can select *Wolbachia* variants that increase host resistance and counter‐selection variants that increase host susceptibility to infection, thereby potentially driving the spread of defensive *Wolbachia* variants (e.g., Cattel, Martinez, Jiggins, Mouton, & Gibert, 2016; Jaenike, Unckless, Cockburn, Boelio, & Perlman, 2010; Kriesner & Hoffmann, 2018). Hence, assessing the effect of natural *Wolbachia* infection on the efficiency of different strains and/or species of parasitic biocontrol agents is a prerequisite for the development of efficient and long‐lasting control strategies (Zindel, Gottlieb, & Aebi, 2011).

Spider mites of the genus *Tetranychus* (Acari: Tetranychidae) are ubiquitous major crop pests of c.a. 1,100 plant species belonging to more than 140 different plant families (Migeon & Dorkeld, 2006). Due to their short generation time and high fecundity, spider mites rapidly develop resistance to pesticides (Van Leeuwen, Vontas, Tsagkarakou, Dermauw, & Tirry, 2010), which has encouraged the development of alternative control strategies such as the use of essential oils or natural enemies (e.g., predators, entomopathogenic bacteria and fungi; Attia et al., 2013). Among them, entomopathogenic fungi have been successfully used in integrated pest management (IPM) programs, and commercial formulations are currently available to farmers in most parts of the world (Skinner, Parker, & Kim, 2014). In particular, fungi such as *Beauveria bassiana*, *Metarhizium* spp., *Isaria* spp. and *Lecanicillium* spp. have been identified as good candidates for efficient spider mite control (e.g., Bugeme, Maniania, Knapp, & Boga, 2008; Chandler, Davidson, & Jacobson, 2005; Maniania, Bugeme, Wekesa, Delalibera, & Knapp, 2008; Shin, Bae, Kim, Yun, & Woo, 2017), and their compatibility with other control methods, such as predatory mites (e.g., Dogan, Hazir, Yildiz, Butt, & Cakmak, 2017; Ullah & Lim, 2017; Wu, Xie, Li, Xu, & Lei, 2016) or pesticides (e.g., Klingen & Westrum, 2007; Shi, Jiang, & Feng, 2005) is widely studied. Curiously, however, the interaction between entomopathogenic fungi and bacterial endosymbionts of spider mites has, to our knowledge, never been investigated. This is at odds with the fact that, on the one hand, natural populations of spider mites often carry several maternally inherited endosymbiotic bacteria with variable prevalence, *Wolbachia* being the most prevalent (prevalence ranges from 0% to 100%; e.g. Gotoh, Sugasawa, Noda, & Kitashima, 2007; Zélé, Santos, et al., 2018; Zhang, Chen, Yang, Qiao, & Hong, 2016); and, on the other hand, *Wolbachia* has been shown to protect *Drosophila melanogaster* hosts against the mortality induced by *B. bassiana* (Panteleev et al., 2007), although no such effect has been found in *D. simulans*; (Fytrou, Schofield, Kraaijeveld, & Hubbard, 2006).

To examine the effect of the interaction between *Wolbachia* and fungal infection on spider mite survival, we carried out a fully factorial experiment using two naturally *Wolbachia*‐infected and two naturally *Wolbachia*‐uninfected spider mite populations belonging to two genetically differentiated forms of *T. urticae* (Auger, Migeon, Ueckermann, Tiedt, & Navajas Navarro, 2013) and treated or not with antibiotics. We used a strain of two generalist entomopathogenic fungi species, *Beauveria bassiana* and *Metarhizium brunneum*, as they are included in genuses that are among the most used fungi in commercial production (Vega et al., 2009), with wide geographical and host ranges (Gurlek, Sevim, Sezgin, & Sevim, 2018; Meyling & Eilenberg, 2007; Roberts & Leger, 2004). The specific aims of this work were to determine: (a) whether infection with a natural *Wolbachia* strain protects spider mites against fungus‐induced mortality, (b) whether this effect varies with different *Wolbachia* strains and/or the presence of other bacteria in spider mites, and (c) whether this effect depends on the fungus strain. We then discuss possible mechanisms leading to our results, the importance of considering the whole bacterial community of arthropods when assessing the effect of *Wolbachia*, as well as the potential eco‐evolutionary consequences of the presence of *Wolbachia* for the success of spider mite control strategies using entomopathogenic fungi.

## 2 MATERIALS AND METHODS

### 2.1 Spider mite populations and rearing

Four populations were used in this study, two belonging to the “red” form (AlRo and AMP), and two belonging to the “green” form (DEF and TOM) of *Tetranychus urticae* (Auger et al., 2013). These populations have been collected in the Iberian Peninsula from 2010 to 2017, on different plant species. Upon collection from the field, the populations AMP and TOM were found to be naturally and fully infected by two different strains of *Wolbachia*. The population AMP is infected by the *Wolbachia* strain ST481 (isolate “Turt_B_wUrtAmp,” id: 1858 in the PubMLST *Wolbachia* database; http:// http://www.pubmlst.org/wolbachia/), which is very similar to strain ST219 belonging to supergroup B and found in China by Zhang, Ding, Zhang, and Hong (2013); and the population TOM is infected by the *Wolbachia* strain ST280 (isolate “Turt_B_wUrtTom,” id: 1857), which has also been previously found in China by Zhang, Ding, et al. (2013). These strains are very closely related, having 1 SNP difference on the sequences of both the fbpA and coxA genes in the multilocus sequence typing (MLST) system developed by Baldo et al. (2006) for *Wolbachia*. The two other populations, AlRo and DEF, were naturally uninfected by *Wolbachia* and none of the populations used in this study carried other maternally inherited bacterial endosymbionts (i.e., *Cardinium*, *Rickettsia, Spiroplasma, and Arsenophonus*) at the time of the experiment, as confirmed by PCR using the methods described in Zélé, Santos, et al. (2018). All the information concerning these populations is summarized in Table 1. After collection, these populations were reared in the laboratory under standard conditions (24 ± 2°C, 60% RH, 16/8h L/D) at high numbers (c.a. 500–1000 females per population) in insect‐proof cages containing bean plants (*Phaseolus vulgaris*, cv. Contender seedlings obtained from Germisem, Oliveira do Hospital, Portugal).

**Table 1:** Populations of spider mites used in the experiment. Mites were collected in Portugal (P) and Spain (S) and were naturally infected, or not, by *Wolbachia*. The absence of other maternally inherited endosymbionts (*Cardinium*, *Rickettsia, Spiroplasma, Arsenophonus*) in these populations was confirmed by PCR before the onset of the experiment (using methods described in Zélé, Santos, et al., 2018; Zélé, Weill, et al., 2018)

Name	Date	Host plant	Location	Coordinates	*Wolbachia* infection	Reference
AlRo	09/11/2013	*Rosa spp.*	Almería (S)	36.855725, −2.320374	no	(Zélé, Santos, et al., 2018)
DEF	26/04/2017	*Solanum lycopersicum*	Alvalade, Lisbon (P)	38.75515, −9.14685	no	–
AMP	18/11/2013	*Datura stramonium*	Aldeia da Mata Pequena (P)	38.534363, −9.191163	yes (ST481^a^)	(Zélé, Santos, et al., 2018)
TOM	‐‐/05/2010	*Solanum lycopersicum*	Carregado (P)	39.078962,−8.993656	yes (ST280^b^)	(Clemente, Rodrigues, Ponce, Varela, & Magalhães, 2016)

a Isolate “Turt_B_wUrtTom”—id: 1857, *Wolbachia* strain ST280. This strain has been first identified as wTurt_2 from three different populations of *T. urticae* in China (Zhang, Zhang, et al., 2013).

b Isolate “Turt_B_wUrtAmp”—id: 1858, *Wolbachia* strain ST481. This is a new strain, very similar to the strain ST219 (they differ by 1 SNP on the fbpA gene: allele 444 instead of allele 4) that was found in China by Zhang, Ding, et al. (2013).

### 2.2 Antibiotic treatments

Roughly 2 months (ca. 4 generations) before the onset of the experiment, a rifampicin solution (0.05%, w/v) was used to treat mites (*n* = 70 adult females initially) from each population for one generation (see Gotoh et al., 2005). This allowed us to obtain *Wolbachia*‐uninfected AMP and TOM populations as well as controls for the antibiotic treatment for the naturally uninfected populations AlRo and DEF. During the treatment, mites were maintained in Petri dishes containing bean leaf fragments placed on cotton with the antibiotic solution. After one generation, 100 adult‐mated daughters from each treated population were transferred in insect‐proof cages containing bean plants, in the same laboratory conditions as the untreated populations, and these new populations were allowed to grow for 3 successive generations in the absence of antibiotics to avoid potential side effects of the treatment (e.g., Ballard & Melvin, 2007). One generation before the onset of the experiment, pools of 100 females were taken from each treated population and checked by PCR to confirm that they were uninfected by *Wolbachia* (detailed procedure in Zélé, Weill, & Magalhães, 2018). This method allows detecting *Wolbachia* infection even at low frequencies (up to 1/100; Zélé, Weill, et al., 2018).

### 2.3 Entomopathogenic fungi strains and preparation of inoculum

We used the strains V275 (= Met52, F52, BIPESCO 5) of *Metarhizium brunneum* and UPH‐1103 of *Beauveria bassiana* (obtained from Swansea University; UK, and from Siedlce University; Poland, respectively), as they were previously shown to have the potential to suppress *T. urticae* populations (Dogan et al., 2017). The procedures used for fungal growth, inoculum preparation, and spider mite infection are similar to that described in Dogan et al. (2017). Briefly, the two fungi were grown on Sabouraud Dextrose Agar (SDA) medium at 25°C for 2 weeks. Conidia were harvested from sporulating cultures with the aid of a spatula, washed with sterile distilled water and filtered through 4 layers of gauze (pore size: 20 µm) to remove any hyphae.

### 2.4 Spider mite infection and survival

The experiment was conducted in a growth chamber under standard conditions (25 ± 2°C, 80% RH, 16/8 hr L/D). Roughly 2 weeks prior to the experiment, 100 females were collected from each mass culture and allowed to lay eggs during 4 days on detached bean leaves placed on water‐soaked cotton. One day prior to the onset of the experiment, 20 young adult mated females (hence with similar age) were randomly collected from these cohorts and placed on a 9 cm^2^ bean leaf disc on wet cotton with the abaxial surface facing upwards. On the first day of the experiment, the surface of the leaf discs was sprayed using a hand sprayer with 2.5 ml of a spore suspension of *M. brunneum* or *B. bassiana* in 0.03% (v/v) aqueous Tween 20 at 1 × 10^7^ conidia/ml (which is the most commonly used concentration in laboratory studies; Dogan et al., 2017), or, as a control, with 0.03% aqueous Tween 20 only. Subsequently, female survival was monitored every 24 hr during 10 days by counting both dead and alive individuals. A total of twelve replicates per treatment (fungi infection and antibiotic treatment) and per population were performed within 2 experimental blocks of one day difference (6 replicates of each treatment per block).

### 2.5 Statistical analysis

Analyses were carried out using the R statistical package (version 3.5.3). The general procedure for building the statistical models was as follows. Spider‐mite populations (AlRo, AMP, DEF, and TOM), antibiotic treatment (treated with rifampicin or not), and infection treatment (sprayed with BB: *Beauveria bassiana*, with MB: *Metarhizium brunneum*, or with Tween 20 only as control) were fitted in as fixed explanatory variables, whereas discs nested within population and block were fitted as random explanatory variables. When a significant three‐way interaction between the three fixed variables was found, each population was analyzed separately with the same model structure, except that the variable population was removed from the model.

Survival data were analyzed using Cox proportional hazards mixed‐effect models (coxme, kinship package). Hazard ratios (HR) were obtained from these models as an estimate of the difference between the rates of dying (Crawley, 2007) between the untreated controls and the BB or MB treatments for each population. Because the timing of infection is an important parameter for the fitness of parasites, an additional early measurement of survival, the proportion of dead mites at 3 days postinfection (dpi), was obtained from Kaplan–Maier estimates of the survival distribution for each disc. This timing was chosen as it is close to the median survival upon infection in most of the populations tested, and hence corresponds to a threshold time‐point to unravel differences between treatments. The numbers of dead and alive mites at 3 dpi were computed using the function cbind and analyzed with a mixed model glmmadmb procedure (glmmADMB package) with a negative binomial error distribution to correct for overdispersed errors (family “nbinom1” with a Øµ variance).

Maximal models, including all higher‐order interactions, were simplified by sequentially eliminating non‐significant terms and interactions to establish a minimal model (Crawley, 2007). The significance of the explanatory variables was established using chi‐squared tests (Bolker, 2008). The significant chi‐squared values given in the text are for the minimal model, whereas nonsignificant values correspond to those obtained before deletion of the variable from the minimal model.

To explore significant interactions between infection and antibiotic treatment effects on female survival and mortality at 3 dpi, the two factors were concatenated to fit a single fixed factor containing all treatment levels in the models (i.e., 6 levels for infection by antibiotic treatment effects within each population). Multiple comparisons between levels were then performed from these models using General Linear Hypotheses (glht, package multicomp) with Bonferroni corrections, which uses classical Chisq (Wald test) for testing the global hypothesis *H_0_*.

## 3 RESULTS

Overall, depending on whether they were naturally infected or uninfected by *Wolbachia*, the survival of females from different populations was not evenly affected by fungal infection and by rifampicin treatment (*fungal infection x rifampicin treatment x population* interaction: *X^2^_6_* = 36.16, *p* < .0001 and *X^2^_6_* = 18.33, *p* = .005 on the overall survival of spider mites and on their mortality at 3 days postinfection, respectively). Thus, to understand this three‐way interaction, we looked at the effect of fungal infection and of the antibiotic treatment in each population separately.

### 3.1 Effect of fungal infection and of antibiotic treatment in the naturally *Wolbachia*‐uninfected population AlRo

**Figure 1:**
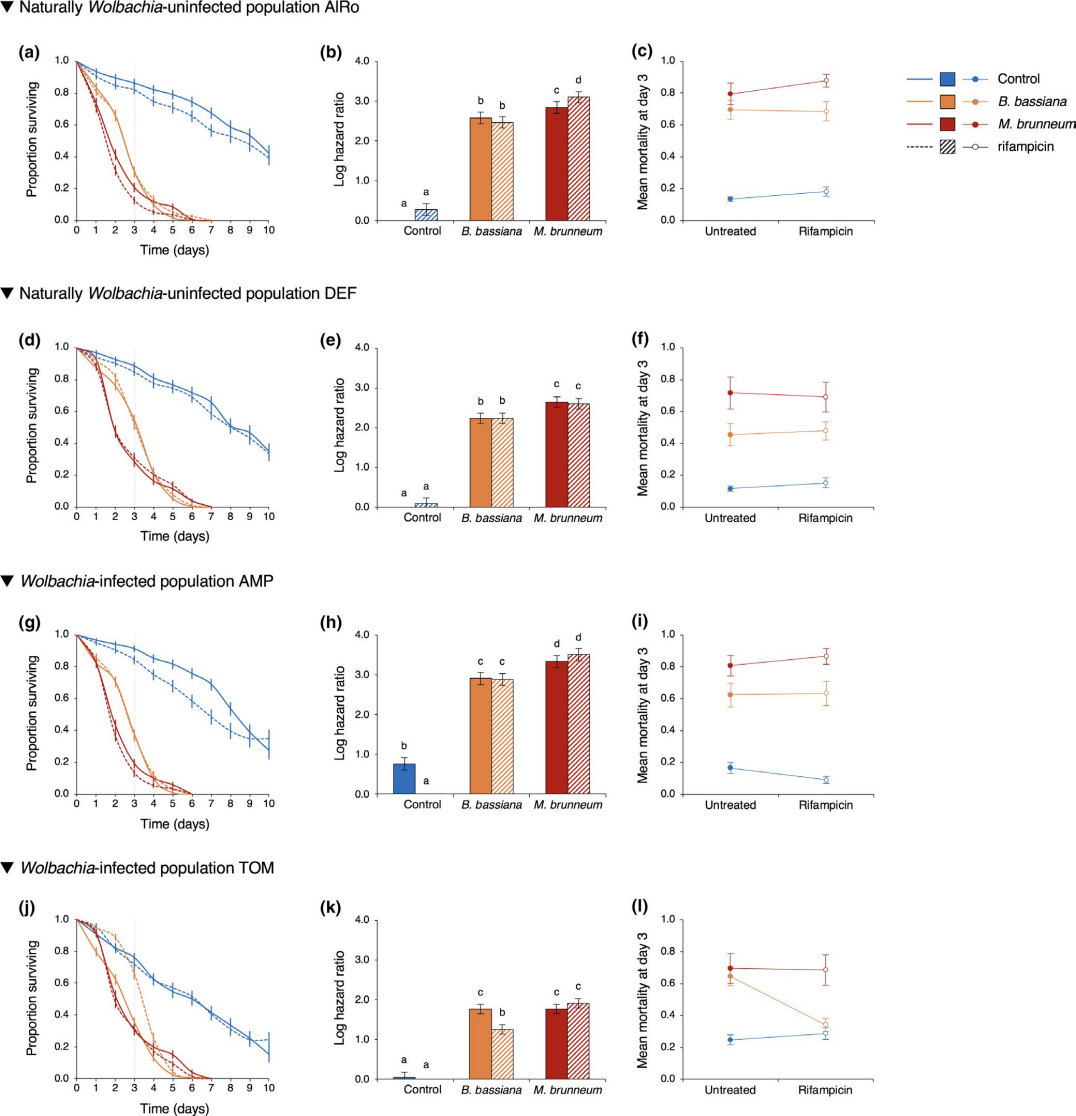
Survival curves (proportion surviving ± s.e.) (a,d,g,j), relative mortality (estimated log hazard ratio ± s.e.) (b,e,h,k), and average survival (± s.e.) at 3 dpi (c,f,i,l) of spider mites from the naturally *Wolbachia*‐uninfected populations AlRo (a,b,c) and DEF (d,e,f), and the naturally *Wolbachia*‐infected populations AMP (g,h,i) and TOM (j,k,l). Adult females were treated (dashed lines, dashed bars, and empty circles) or not (solid lines, filled bars, and circles) with rifampicin, and sprayed with *B. bassiana* (orange), *M. brunneum* (red), or Tween 20 only as control (blue)

In the population AlRo, the two fungal strains affected differently the survival of spider mites depending on whether they were treated with antibiotics or not (*fungal infection x rifampicin treatment* interaction: *X^2^_2_* = 9.53, *p* = .009; Figure 1a). Indeed, *M. brunneum* (MB) induced a stronger mortality in rifampicin‐treated mites than in untreated mites (z = 2.80, *p* = .05), while *B. bassiana* (BB) induced the same mortality in both rifampicin‐treated and untreated mites (z = −1.20, *p* = 1.00 Figure 1b; Table 2a). In both cases, however, *M. brunneum* induced a stronger mortality than *B. bassiana* (MB vs. BB: z = 6.72, *p* < .0001 and z = 2.81, *p* = .05 in rifampicin‐treated and untreated mites, respectively). At 3 days postinfection (dpi), however, no significant interaction between fungal infection and rifampicin treatment (*X^2^_2_* = 1.78, *p* = .41; Figure 1c) and no effect of the antibiotics treatment alone (*X^2^_1_* = 0.90, *p* = .34) on female mortality were found. Both fungi strains severely increased the mortality of both rifampicin‐treated and rifampicin‐untreated mites at this early age of infection (*X^2^_2_* = 135.68, *p* < .00014; see Table 3a for all multiple comparisons).

**Table 2:** Results of multiple comparisons (with Bonferroni correction) between hazard ratios obtained for the naturally *Wolbachia*‐uninfected populations (a) AlRo, and (b) DEF, and for the naturally *Wolbachia*‐infected populations (c) AMP, and (d) TOM sprayed or not with fungi (BB: *Beauveria bassiana*; MB: *Metarhizium brunneum*; Control: Tween 20 only) and treated or not with antibiotics (rif: rifampicin‐treated; nt: untreated)

(a) Naturally *Wolbachia*‐uninfected population AlRo						
Treatments compared			Estimate	Std. Error	z value	*p*‐value
Control_rif	versus	Control_nt	0.272	0.146	1.869	.554
BB_rif	versus	BB_nt	−0.110	0.092	−1.200	1.000
MB_ rif	versus	MB_nt	0.259	0.093	2.802	.046^a^
BB_ nt	versus	Control_nt	2.578	0.144	17.931	<2e‐16^a^
MB_nt	versus	Control_nt	2.840	0.144	19.718	<2e‐16^a^
MB_ nt	versus	BB_nt	0.262	0.093	2.810	.045^a^
BB_ rif	versus	Control_rif	2.195	0.134	16.345	<2e‐16^a^
MB_rif	versus	Control_rif	2.827	0.139	20.410	<2e‐16^a^
MB_ rif	versus	BB_rif	0.632	0.094	6.718	1.66E‐10^a^

(b) Naturally *Wolbachia*‐uninfected population DEF						
Treatments compared			Estimate	Std. Error	z value	*p*‐value
Control_rif	versus	Control_nt	0.091	0.141	0.648	1.000
BB_rif	versus	BB_nt	−0.001	0.092	−0.015	1.000
MB_ rif	versus	MB_nt	−0.044	0.091	−0.487	1.000
BB_ nt	versus	Control_nt	2.238	0.136	16.435	<2e‐16^a^
MB_nt	versus	Control_nt	2.647	0.138	19.240	<2e‐16^a^
MB_ nt	versus	BB_nt	0.410	0.096	4.246	1.96E‐04^a^
BB_ rif	versus	Control_rif	2.145	0.132	16.220	<2e‐16^a^
MB_rif	versus	Control_rif	2.512	0.134	18.685	<2e‐16^a^
MB_ rif	versus	BB_rif	0.367	0.095	3.845	.001^a^

(c) Naturally *Wolbachia*‐infected population AMP						
Treatments compared			Estimate	Std. Error	z value	*p*‐value
Control_rif	versus	Control_nt	−0.756	0.154	−4.916	7.93E‐06^a^
BB_rif	versus	BB_nt	−0.024	0.091	−0.258	1.000
MB_ rif	versus	MB_nt	0.172	0.092	1.875	.547
BB_ nt	versus	Control_nt	2.151	0.136	15.819	<2e‐16^a^
MB_nt	versus	Control_nt	2.581	0.136	18.921	<2e‐16^a^
MB_ nt	versus	BB_nt	0.430	0.093	4.599	3.81E‐05^a^
BB_ rif	versus	Control_rif	2.883	0.150	19.195	<2e‐16^a^
MB_rif	versus	Control_rif	3.508	0.154	22.828	<2e‐16^a^
MB_ rif	versus	BB_rif	0.625	0.093	6.702	1.85E‐10^a^

(d) Naturally *Wolbachia*‐infected population TOM						
Treatments compared			Estimate	Std. Error	z value	*p*‐value
Control_rif	versus	Control_nt	−0.050	0.130	−0.385	1.000
BB_rif	versus	BB_nt	−0.510	0.092	−5.539	2.74E‐07^a^
MB_ rif	versus	MB_nt	0.145	0.092	1.579	1.000
BB_ nt	versus	Control_nt	1.711	0.120	14.237	<2e‐16^a^
MB_nt	versus	Control_nt	1.715	0.120	14.302	<2e‐16^a^
MB_ nt	versus	BB_nt	0.004	0.093	0.045	1.000
BB_ rif	versus	Control_rif	1.250	0.118	10.603	<2e‐16^a^
MB_rif	versus	Control_rif	1.909	0.121	15.839	<2e‐1^a^
MB_ rif	versus	BB_rif	0.659	0.096	6.878	5.48E‐11^a^

a * *p*‐value < .05, ** *p*‐value < .01, *** *p*‐value < .001.

**Table 3:** Results of multiple comparisons (with Bonferroni correction) between mortality at 3 dpi of the naturally *Wolbachia*‐uninfected populations (a) AlRo, and (b) DEF, and for the naturally *Wolbachia*‐infected populations (c) AMP, and (d) TOM sprayed or not with fungi (BB: *Beauveria bassiana*; MB: *Metarhizium brunneum*; Control: Tween 20 only) and treated or not with antibiotics (rif: rifampicin‐treated; nt: untreated)

(a) Naturally *Wolbachia*‐uninfected population AlRo						
Treatments compared			Estimate	Std. Error	z value	*p*‐value
Control_rif	versus	Control_nt	0.327	0.236	1.387	1.000
BB_rif	versus	BB_nt	−0.012	0.247	−0.049	1.000
MB_ rif	versus	MB_nt	0.085	0.175	0.489	1.000
BB_ nt	versus	Control_nt	1.684	0.196	8.607	<2e−16^a^
MB_nt	versus	Control_nt	1.818	0.194	9.386	<2e−16^a^
MB_ nt	versus	BB_nt	0.134	0.232	0.579	1.000
BB_ rif	versus	Control_rif	1.345	0.291	4.614	3.55E−05^a^
MB_rif	versus	Control_rif	1.576	0.276	5.702	1.06E−07^a^
MB_ rif	versus	BB_rif	0.232	0.202	1.145	1.000

(b) Naturally *Wolbachia*‐uninfected population DEF						
Treatments compared			Estimate	Std. Error	z value	*p*‐value
Control_rif	versus	Control_nt	0.177	0.328	0.539	1.000
BB_rif	versus	BB_nt	0.089	0.368	0.241	1.000
MB_ rif	versus	MB_nt	−0.020	0.258	−0.077	1.000
BB_ nt	versus	Control_nt	1.255	0.277	4.525	5.44E‐05^a^
MB_nt	versus	Control_nt	1.738	0.265	6.550	5.17E‐10^a^
MB_ nt	versus	BB_nt	0.482	0.339	1.423	1.000
BB_ rif	versus	Control_rif	1.167	0.416	2.806	.045^a^
MB_rif	versus	Control_rif	1.541	0.393	3.925	.001^a^
MB_ rif	versus	BB_rif	0.374	0.304	1.231	1.000

(c) Naturally *Wolbachia*‐infected population AMP						
Treatments compared			Estimate	Std. Error	z value	*p*‐value
Control_rif	versus	Control_nt	−0.644	0.270	−2.390	.152
BB_rif	versus	BB_nt	0.013	0.235	0.056	1.000
MB_ rif	versus	MB_nt	0.070	0.170	0.411	1.000
BB_ nt	versus	Control_nt	1.322	0.178	7.424	1.03E‐12^a^
MB_nt	versus	Control_nt	1.579	0.174	9.088	< 2e‐16^a^
MB_ nt	versus	BB_nt	0.257	0.219	1.175	1.000
BB_ rif	versus	Control_rif	1.979	0.316	6.258	3.50E‐09^a^
MB_rif	versus	Control_rif	2.293	0.305	7.529	4.58E‐13^a^
MB_ rif	versus	BB_rif	0.314	0.195	1.608	.970

(d) Naturally *Wolbachia*‐infected population TOM						
Treatments compared			Estimate	Std. Error	z value	*p*‐value
Control_rif	versus	Control_nt	0.125	0.177	0.706	1.000
BB_rif	versus	BB_nt	−0.637	0.234	−2.717	.059.
MB_ rif	versus	MB_nt	−0.012	0.175	−0.069	1.000
BB_ nt	versus	Control_nt	0.949	0.152	6.239	3.96E‐09^a^
MB_nt	versus	Control_nt	1.024	0.151	6.798	9.56E‐11^a^
MB_ nt	versus	BB_nt	0.075	0.200	0.374	1.000
BB_ rif	versus	Control_rif	0.187	0.250	0.751	1.000
MB_rif	versus	Control_rif	0.887	0.220	4.033	4.95E‐04^a^
MB_ rif	versus	BB_rif	0.699	0.205	3.414	.006^a^

a * *p*‐value < .05, ** *p*‐value < .01, *** *p*‐value < .001.

### 3.2 Effect of fungal infection and of antibiotic treatment in the naturally *Wolbachia*‐uninfected population DEF

In the population DEF, we did not find a significant interaction between fungal infection and rifampicin treatment (*X^2^_2_* = 0.65, *p* = .72; Figure 1d), neither a significant effect of rifampicin treatment (*X^2^_1_* = 0.003, *p* = .96), but only a significant effect of fungal infection (*X^2^_2_* = 879.17, *p* < .0001). Indeed, both fungi induced the same mortality in rifampicin‐treated and in rifampicin‐untreated mites, with an overall stronger effect of *M. brunneum* than of *B. bassiana* (Figure 1e; Table 2b for all multiple comparisons). Similarly, at 3 dpi, no significant interaction between fungal infection and rifampicin treatment (*X^2^_2_* = 0.40, *p* = .82; Figure 1f), neither a significant effect of rifampicin treatment (*X^2^_1_* = 0.14, *p* = .71) was found. As for the population AlRo, only fungal infection affected the spider‐mite survival (*X^2^_2_* = 64.89, *p* < .0001; Table 3b for all multiple comparisons).

### 3.3 Effect of fungal infection and of antibiotic treatment in the naturally *Wolbachia*‐infected population AMP

In the population AMP, we found a significant interaction between infection and rifampicin treatment (*X^2^_2_* = 26.61, *p* < .0001; Figure 1g). This interaction was due to a lower survival of *Wolbachia*‐infected controls compared with rifampicin‐treated controls (z = −4.92, *p* < .0001) only, as *Wolbachia*‐infected and rifampicin‐treated mites had the same overall survival upon infection with each fungal strain (for *B. bassiana*: z = −0.26, *p* = 1.00; for *M. brunneum*: z = 1.88, *p* = .55; Figure 1h and Table 2c). However, accounting for this difference between *Wolbachia*‐infected and rifampicin‐treated controls reveals that, relative to their respective control, both fungi induced higher mortality in rifampicin‐treated mites (HR = 17.87 and HR = 33.39, for BB and MB, respectively) than in *Wolbachia*‐infected ones (HR = 8.60 and HR = 13.21, respectively). A significant interaction between infection and rifampicin treatment was also found at 3 dpi (*X^2^_2_* = 6.5, *p* = .04; Figure 1i). However, this interaction was relatively weak at this time‐point and could not be explained by multiple comparisons between factor levels (i.e., no differences were found between *Wolbachia*‐infected and rifampicin‐treated mites when sprayed with Tween 20 only, *B. bassiana*, or *M. brunneum*; Table 3c for all multiple comparisons).

### 3.4 Effect of fungal infection and of antibiotic treatment in the naturally *Wolbachia*‐infected population TOM

In the population TOM, we also found a significant interaction between infection and rifampicin treatment (*X^2^_2_* = 26.00, *p* < .0001; Figure 1j). In this population, the effect of *B. bassiana* was weaker in rifampicin‐treated (HR = 3.49) than *Wolbachia*‐infected mites (HR = 5.53; z = −5.54, *p* < .0001; Figure 1k and Table 2d), while *M. brunneum* had the same effect in both rifampicin‐treated and nontreated mites (HR = 6.75 and HR = 5.56, respectively; z = 1.58, *p* = 1.00). Moreover, whereas both fungi had the same effect on nontreated mites (MB vs. BB: z = 0.05, *p* = 1.00), *B. bassiana* did not decrease the survival of rifampicin‐treated mites as much as *M. brunneum* (MB vs. BB: z = −6.88, *p* < .0001). This effect was even stronger at 3 dpi (*fungal infection x rifampicin* interaction: *X^2^_2_* = 15.44, *p* < .001). At this time‐point, *B. bassiana* induced the same mortality as *M. brunneum* in *Wolbachia*‐infected mites (BB vs. Control: z = 6.24, *p* < .0001), but did not affect significantly the survival of rifampicin‐treated mites (BB vs. Control: z = 0.75, *p* = 1.00; Figure 1l and Table 3d).

## 4 DISCUSSION

In this study, we found variable effects of infection by *B. bassiana* and *M. brunneum* following antibiotic treatment, depending on the spider mite population and on whether spider mites were naturally infected by *Wolbachia* or not. Indeed, the mortality induced by both fungi did not differ between *Wolbachia*‐infected and rifampicin‐treated mites in the population AMP, despite *Wolbachia* infection being costly in absence of fungal infection. Similarly, the mortality induced by *M. brunneum* was not affected by *Wolbachia* infection in the population TOM, but the mortality induced by *B. bassiana* increased in presence of *Wolbachia*. These results suggest that *Wolbachia* may buffer, or conversely increase, the effect of fungal infection depending on the fungi strains, the *Wolbachia* strain and/or the host genetic background. Moreover, in absence of natural *Wolbachia* infection, we found a relatively small effect of the antibiotic treatment on mite susceptibility to infection: The antibiotic treatment had no effect on the outcome of infection by fungi, with the exception of a higher mortality in rifampicin‐treated mites from the population AlRo when infected with *M. brunneum*. This effect, although significant, is of relatively low amplitude and in the opposite direction than that observed in the *Wolbachia*‐infected population TOM following *B. bassiana* infection. This suggests that the effect of *Wolbachia* in the population TOM may not be explained by an alteration of the whole bacterial community in mites following antibiotic treatment. However, because the effect of fungal infection and antibiotic treatment vary between populations independently of the presence of *Wolbachia*, we draw caution on the generalization of such results.

In different arthropod host species, *Wolbachia* may either protect (e.g., Braquart‐Varnier et al., 2015; Hughes, Koga, Xue, Fukatsu, & Rasgon, 2011; Kambris, Cook, Phuc, & Sinkins, 2009; Moreira et al., 2009; Panteleev et al., 2007; Teixeira, Ferreira, & Ashburner, 2008), have no effect (e.g., Tortosa, Courtiol, Moutailler, Failloux, & Weill, 2008; Wong, Hedges, Brownlie, & Johnson, 2011; Zouache, Michelland, Failloux, Grundmann, & Mavingui, 2012), or even increase the susceptibility (e.g., Graham et al., 2012; Hughes et al., 2014) of its arthropod hosts to infection depending on the pathogens tested, the *Wolbachia* strain (e.g., Chrostek et al., 2013; Martinez et al., 2017; Osborne, Leong, O'Neill, & Johnson, 2009), but also the host genetic background (although to a lesser extent; e.g. Martinez et al., 2017). In several of these studies the effect of *Wolbachia* on host susceptibility to pathogens has been assessed following artificial *Wolbachia* infection (e.g., Joubert et al., 2016; Moreira et al., 2009; Walker et al., 2011), which prevents a direct alteration of the host bacterial community but may not accurately reflect the effect of natural *Wolbachia* infections. Indeed, novel *Wolbachia*‐host associations are often costly for hosts (e.g., McGraw, Merritt, Droller, & O'Neill, 2002), mainly due to the activation of the host immune system, which in turn prevents subsequent infections by other pathogens (reviewed by Zug & Hammerstein, 2015). Conversely, the effect of natural *Wolbachia* infections on host susceptibility to pathogens is usually assessed by using antibiotic treatments. However, antibiotics do not affect *Wolbachia* only, but also the entire bacterial community in hosts (e.g., Lehman, Lundgren, & Petzke, 2009; Zouache, Voronin, Tran‐Van, & Mavingui, 2009), which raises the necessity to assess the effect of the antibiotic treatment per se.

In *T. truncatus* spider mites, Zhu et al. (2018) showed that antibiotic treatment affects the composition of the bacterial community even after more than 20 generations without antibiotics. In particular, bacteria from different families increased in proportion in tetracycline‐treated mites in absence of the Anaplasmataceae (which includes *Wolbachia*). Hence, in our study, the lower mortality observed for antibiotic‐treated mites following infection by *B. bassiana* in the naturally *Wolbachia*‐infected population TOM cannot be unambiguously attributed to *Wolbachia* only. This result could be explained, for instance, by *Wolbachia* outcompeting bacteria that contribute to the host homeostasis and immunity (reviewed by Shapira, 2016; Vavre & Kremer, 2014; Weiss & Aksoy, 2011), thereby increasing the success of *B. bassiana* infection (i.e., indirect facilitation; Zélé, Magalhães, Kéfi, & Duncan, 2018). In contrast, in the *Wolbachia*‐uninfected population AlRo, antibiotic‐treated mites have a higher mortality than untreated mites when infected with *M. brunneum*. One possible explanation is that, in the absence of natural *Wolbachia* infection, the antibiotic treatment affected differently the bacterial community, potentially eliminating bacteria that interfere with *M. brunneum*.

The apparent facilitation of *B. bassiana* by *Wolbachia* in the TOM population may also be due to *Wolbachia* interacting directly with the host immune system. Indeed, *Wolbachia* can downregulate autophagy‐associated genes in naturally infected hosts, possibly as an immune evasion strategy (Chevalier et al., 2012; Kremer et al., 2009). Under such scenario, the elimination of *Wolbachia* with antibiotics may result in overall higher autophagic processes in the host, to which *B. bassiana* could be susceptible. Moreover, in diverse native hosts, including *T. urticae*, *Wolbachia* also plays a role in redox homeostasis (e.g., Zhang, Ding, Rong, & Hong, 2015; Zug & Hammerstein, 2015). The elimination of *Wolbachia* with antibiotics in coevolved *T. urticae* hosts may thus potentially lead to a disruption of redox homeostasis and higher production of reactive oxygen species (ROS), which are involved in host immunity (e.g., encapsulation, melanisation; reviewed by Zug & Hammerstein, 2015), thereby increasing host resistance to infection. However, all these different scenarios would only explain our results if such mechanisms affect differently the two fungal strains and are specific to the *Wolbachia* strain and/or the host population.

As stated above, the host genetic background also plays a major role in determining host susceptibility to infection. First, not all populations (independently of their status of infection by *Wolbachia*) are equally affected by the infection by the two fungi (e.g., the mortality induced by both fungi is stronger in the population DEF than in the population TOM). Indeed, we have previously shown both inter‐ and intraspecific variability in spider mite susceptibility to infection by the same two fungi (Zélé et al. in press). Second, host susceptibility to infection may also result from Genotype x Genotype interactions with their endosymbionts (e.g., Martinez et al., 2017). Here, the different effects of *Wolbachia* observed in the populations TOM and AMP cannot be unambiguously attributed to the *Wolbachia* strain only, but likely result from their interaction with the host genetic background. Hence, although further investigations on the respective role of the *Wolbachia* strain and of the host genome in the susceptibility to different fungal strains (e.g., by using several spider mite populations infected by the same and different *Wolbachia* strains and by different fungal strains belonging to different species), as well as on the composition of the bacterial communities in each of the population tested would be necessary to shed light on the mechanisms involved, these results show that the outcome of infection strongly depends on complex interactions between multiple microorganisms and their host.

Irrespective of the underlying mechanisms, the variable effects of *Wolbachia* on spider mite susceptibility observed here raise important questions about the potential consequences of the use of biocontrol agents for both the ecology and epidemiology of naturally occurring *Wolbachia* infection in arthropod pests. Indeed, the artificial introduction of pathogens for biocontrol may counter‐select susceptible *Wolbachia*‐host combinations and potentially select for defensive *Wolbachia* variants, leading to their spread across host populations (e.g., Cattel et al., 2016; Jaenike et al., 2010; Kriesner & Hoffmann, 2018). A better understanding of the variability in the outcome of *Wolbachia*‐host–pathogens interactions, as well as its consequences for the ecology and evolution of all players of such interaction, is thus a challenge with both fundamental and applied interests, and future work should go in that direction.

In conclusion, our results show variable effects of *Wolbachia* on spider mite susceptibility to fungi‐induced mortality using two generalist fungi, *B. bassiana* and *M. brunneum*. To our knowledge, this is the first study investigating the interaction between natural *Wolbachia* infections and widely used biocontrol agents. As *Wolbachia* was found to have either no effect or to increase spider mite susceptibility to fungal infection, these results suggest that it may improve the success of biological control using entomopathogenic fungi. However, these results also highlight the complexity of within‐host–pathogens interaction and caution against the generalization of such effects as (a) the outcome of these interactions may vary depending on the fungal strain, the *Wolbachia* strain, and the host genetic background, and (b) these interactions may evolve at a rapid pace with potentially important consequences for the ecology and epidemiology of *Wolbachia* infection in arthropod pests. Finally, our findings also point to the importance of considering the whole bacterial community of arthropods when assessing the effect of *Wolbachia* in a particular system.

## CONFLICT OF INTEREST

None declared.

## AUTHORS’ CONTRIBUTIONS

FZ and SM conceived and designed the experiment; IS was involved in the maintenance of spider mite populations and plants; MA acquired the data; FZ performed statistical analyses; FZ and SM wrote the manuscript, with input from all authors; IC and SM funded the study. All authors have read and approved the final version of the manuscript.

## Data Availability

Full dataset has been deposited in the Dryad data repository (doi.org/10.5061/dryad.9p8cz8wc4).
